# Silver Nanoparticle-Functionalized Medical Devices for Primary Prevention of Healthcare-Associated Infections: In Vitro Efficacy Against Multidrug-Resistant Clinical Isolates

**DOI:** 10.3390/microorganisms14071534

**Published:** 2026-07-14

**Authors:** Daniela Chirizzi, Rebecca Pellegrino, Federica Paladini, Fabiana D’Urso, Mauro Pollini, Francesco Broccolo

**Affiliations:** 1 UOSD Virology and Microbiology, University Hospital, P.O. Vito Fazzi, ASL Lecce, 73100 Lecce, Italy; 2 Department of Engineering for Innovation, University of Salento, 73100 Lecce, Italy; rebecca.pellegrino@unisalento.it; 3 Department of Experimental Medicine, University of Salento, 73100 Lecce, Italy; federica.paladini@unisalento.it (F.P.); fabiana.durso@unisalento.it (F.D.); mauro.pollini@unisalento.it (M.P.)

**Keywords:** silver nanoparticles, healthcare-associated infections, antimicrobial resistance, biofilm, medical devices, HEPA filters, catheters, multidrug resistant pathogens, photoreduction, nanomedicine

## Abstract

Healthcare-associated infections represent a global public health challenge, primarily driven by biofilm-forming multidrug-resistant pathogens on medical device surfaces, leading to high morbidity, mortality, and healthcare costs. As antibiotic strategies fail due to antimicrobial resistance, this study investigates a primary-prevention nanotechnology strategy based on the photochemical functionalization of clinically relevant substrates (polyurethane venous catheters, cotton gauze, and glass-fiber HEPA filter membranes) with immobilized silver nanoparticles. While the photochemical deposition ensures a controlled silver release experimentally quantified here by ICP-MS (≈0.44 ppm·day^−1^) within safety thresholds, the core value of this functionalization lies in its broad-spectrum and long-term efficacy. Agar diffusion assays performed against a panel of 10 multidrug-resistant clinical isolates, including pathogens such as *Pseudomonas aeruginosa*, *Klebsiella pneumoniae*, *Staphylococcus epidermidis*, *Candida parapsilosis*, and *Aspergillus sydowii*, demonstrated antimicrobial activity over 15 days across all substrates. Biofilm quantification by Crystal Violet Assay revealed inhibition exceeding 97% for bacterial strains and 96% for fungal species at day 15. By preventing both bacterial and fungal colonization on diverse materials, these results validate silver-functionalized surfaces as an effective and bio sustainable approach to combat healthcare-associated infections and antimicrobial resistance. This strategy holds significant promise for translation into clinical settings, hospital air filtration systems, and wound care applications, aligning with Antimicrobial Stewardship and One Health principles.

## 1. Introduction

Healthcare-associated infections (HAIs), also known as nosocomial or hospital-acquired infections, constitute one of the most critical challenges facing contemporary medicine. According to the World Health Organization (WHO) and the European Centre for Disease Prevention and Control (ECDC), HAIs are defined as infective complications arising during the course of healthcare in a medical facility that were neither present nor incubating at the time of patient admission [1,2]. Conventionally, infections manifesting more than 48 h after admission or within 30 days of discharge are classified as HAIs. Epidemiological data from the ECDC 2022–2023 point-prevalence survey confirm a mean HAI prevalence of 7.1% in EU/EEA acute-care hospitals, translating into approximately 3.8 million affected patients annually and over 110,000 attributable deaths [3].

The four dominant HAI phenotypic categories—catheter-associated urinary tract infections (CAUTI), ventilator-associated pneumonia (VAP), surgical site infections (SSI), and central line-associated bloodstream infections (CLABSI)—collectively account for approximately 70–80% of the global infective burden [1,2]. Italy occupies a particularly vulnerable position, with a mean HAI prevalence of 8.8% and among the highest rates of carbapenem-resistant *Acinetobacter baumannii* and vancomycin-resistant *Enterococcus faecium* in Europe [4].

A central pathogenic mechanism underlying device-related HAIs is the formation of microbial biofilm on abiotic surfaces of invasive biomaterials such as intravascular catheters, endotracheal cannulas, prostheses, and drainage systems, as well as on non-invasive materials including wound dressings and air filtration membranes [5]. Because complete biofilm eradication is often clinically unfeasible without device removal, prevention of primary adhesion through functionalized surfaces represents the preferred strategy [6].

The global spread of antimicrobial resistance (AMR) further exacerbates this scenario. Lancet data estimate that in 2019 alone, approximately 4.95 million deaths were associated with bacterial AMR infections, a figure projected to reach 1.91 million direct annual deaths by 2050 [7,8]. Pharmaceutical environmental pollution-antibiotic residues dispersed in hospital effluents exert constant selective pressure favoring horizontal gene transfer (HGT) of resistance determinants between clinical and environmental microorganisms [7]. Reducing systemic antibiotic dependence through device-level prevention thus becomes a core objective of both Antimicrobial Stewardship and One Health frameworks.

Silver nanoparticles (AgNPs) have emerged as one of the most promising nanotechnological solutions for HAI prevention, exhibiting a broad-spectrum antimicrobial activity encompassing MDR bacteria, fungi, and viruses [9,10]. Unlike conventional antibiotics that typically target a single metabolic pathway, thereby favoring resistance emergence, AgNPs exert a pleiotropic cytotoxic action involving simultaneous disruption of membrane integrity (contact killing), induction of reactive oxygen species (ROS), inhibition of ATP-synthase and the electron transport chain via binding to thiol (-SH) groups, interference with DNA replication, and inhibition of biofilm formation through quorum sensing disruption [11,12,13]. This multidimensional attack mechanism dramatically reduces the probability of cross-resistance emergence [14].

In contrast to conventional colloidal silver formulations characterized by uncontrolled ionic release and associated cytotoxicity risks, immobilized AgNPs produced by photochemical deposition (photoreduction) demonstrate a minimally diffusive ionic release profile [15,16]. Existing silver-based device coatings illustrate both the promise and the limitations of this strategy. Ionic- and colloidal-silver catheters, silver-sulfadiazine dressings and related products have reduced device-related infection rates in clinical studies; however, they are commonly constrained by a rapid “burst” and poorly controlled Ag^+^ release that depletes the silver reservoir within days, by dose-dependent cytotoxicity and argyria risk, by a consequent loss of long-term efficacy, and by leaching that contributes to ecotoxicity and to efflux-mediated bacterial resistance against free silver ions [15,16]. The immobilized, minimally diffusive AgNPs employed in the present work are designed specifically to address these shortcomings, privileging surface contact-killing over uncontrolled ionic diffusion and thereby preserving activity over the device service life. UV irradiation of silver nitrate precursor serves as a clean reducing agent enabling tight control over nucleation and crystal growth, yielding monodisperse nanoparticles directly anchored to polymeric and textile matrices with high mechanical stability and without toxic residual reagents [15,17]. This technology has previously been validated on hemodialysis catheters, Foley catheters, and cotton gauze for antibacterial applications [15,16,18]; however, comprehensive multi-substrate evaluation against a clinically representative MDR panel, including both bacterial and fungal pathogens, with integrated biofilm kinetic profiling and long-term safety assessment has not yet been reported. Specifically, whereas the photochemical platform has so far been documented on individual substrates in isolation—hemodialysis catheters [15], Foley catheters [16] and cotton gauze [18]—the present work is the first to apply and cross-validate it simultaneously across three distinct device classes (intravascular, wound-care and air-filtration) against a single, clinically representative ten-strain MDR bacterial and fungal panel, coupling 15-day antimicrobial persistence with 30-day biofilm-kinetic profiling.

The present study addresses this gap by validating the photochemical AgNPs functionalization of three distinct clinical substrates (polyurethane venous catheters, hydrophilic cotton gauzes, and HEPA-class fiber-glass filter membranes) against a panel of 10 MDR clinical isolates. The specific objectives were: (i) assessment of the antimicrobial and antifungal activity by agar diffusion and microbial viability recovery assays over 15 days; and (ii) quantitative determination of biofilm inhibition kinetics by the Crystal Violet assay over 30 days. The aim is to position this nanotechnology within Circular Nanomedicine and Planetary Health paradigms as a concrete primary-prevention strategy against HAIs.

## 2. Materials and Methods

### 2.1. Materials

Silver nitrate (AgNO_3_, 99+%) was purchased from Alfa Aesar (Fisher Scientific, Waltham, MA, USA); Tryptic Soy Broth (TSB), methanol (≥99.9%, MW 32.04) were purchased from Sigma Aldrich (Saint Louis, MO, USA); Tryptic soy agar (TSA) was purchased from Lab Logistics Group GmbH (Meckenheim, Germany); Columbia Agar supplemented with 5% sheep blood (COS), CAN2 chromogenic agar (Candida) and Sabouraud Dextrose Agar (SDA) plates were purchased from bioMérieux (Marcy l′Etoile, France). All aqueous solutions were prepared using distilled water.

### 2.2. Treatment of Three Clinical Substrates

The experimental study evaluated three clinically relevant substrates: dual-lumen polyurethane venous catheters, hydrophilic cotton gauzes, and glass-fiber HEPA filter membranes. The functionalization was performed using a patented photochemical technique that enables the in situ deposition of AgNPs directly onto the substrate surface from a silver salt dissolved in an alcohol [19]. This method, previously validated for the functionalization of different materials, is based on the photo-reduction of AgNO_3_ to metallic AgNPs using methanol as both solvent and reducing agent. Briefly, a 4% *w*/*w* AgNO_3_ solution in methanol was prepared, and the substrates were immersed for 5 min. For catheter samples, the solution was actively injected using a syringe to ensure complete internal luminal coating. Subsequently, the samples were exposed to UV irradiation (1000 W, 20 cm distance; Jelosil, Milano, Italy) for 30 min to induce reduction of silver ions (Ag^+^) and promote AgNPs formation. Finally, the functionalized substrates were thoroughly rinsed with distilled water to remove any residual unreacted precursors.

### 2.3. Physicochemical and Morphological Characterization of Functionalized Substrates

Deposition on the present batch of treated substrates was confirmed by scanning electron microscopy (SEM; Zeiss EVO, Oxford Instruments, Oxford, UK, and JEOL JSM-6500F) with secondary- and backscattered-electron imaging, energy-dispersive X-ray spectroscopy (EDX; Bruker Corporation, Billerica, MA, USA), and transmission electron microscopy (TEM; Tecnai G2, FEI Company, Hillsboro, OR, USA, operated at 300 kV) on carbon-coated Cu grids functionalized with the identical immersion/UV protocol. Surface elemental composition was quantified by EDX before and after a 30-day intensive washing protocol (deionized water recirculated at 37 °C, 5 L·min^−1^, peristaltic pump) to probe coating adhesion under mechanical/hydrodynamic stress. Silver-ion (Ag^+^) release kinetics were determined experimentally for the present study by inductively coupled plasma mass spectrometry (ICP-MS; iCAP-Q, Thermo Fisher, Bremen, Germany): 3 cm catheter segments were incubated in phosphate-buffered saline (PBS, pH 7.4) at 37 °C and aliquots assayed at 1, 3, 7, 10, 14, and 30 days against transition-element CCS-6 standards (1–100 ppb). 

### 2.4. Microbial Panel and Characterization

Clinical isolates were collected at P.O. Vito Fazzi between July 2025 and February 2026, representative of the main HAI etiologies, and rendered fully anonymous after routine diagnostic testing per institutional guidelines. The panel comprised: Gram-negative bacteria—*Pseudomonas aeruginosa* MDR (carbapenem- and fluoroquinolone-resistant, from VAP) and *Klebsiella pneumoniae* carbapenem-resistant (genotypes KPC, NDM, OXA-48); Gram-positive bacteria—*Staphylococcus epidermidis* (biofilm-producing, from CLABSI), *Staphylococcus aureus* (MRSA and MSSA), and *Enterococcus faecium*; and fungi—*Candida albicans*, *Candida tropicalis*, *Candida parapsilosis*, *Aspergillus sydowii*, and *Penicillium* spp. All strains (minimum three per species) were identified by MALDI-TOF mass spectrometry (Vitek MS, bioMérieux), resistance determinants confirmed by GeneXpert molecular platform, and drug-susceptibility profiles established with Vitek 2. Strains were cryopreserved at −80 °C in 20% glycerol. The complete antimicrobial-susceptibility profiles (MIC/category by Vitek 2, EUCAST breakpoints) and the molecular resistance determinants of every isolate—including the carbapenem- and fluoroquinolone-resistant phenotype of *P. aeruginosa*, the KPC/NDM/OXA-48 genotypes of carbapenem-resistant *K. pneumoniae*, the MRSA/MSSA status of *S. aureus*, and the azole-susceptibility of the *Candida* spp.

### 2.5. Agar Diffusion Assay

Antimicrobial activity was assessed by the agar diffusion method (SNV 195920-1992). For catheters, TSA plates were inoculated with 0.5 McFarland suspensions (≈1.5 × 10^8^ CFU/mL for bacteria; 1–5 × 10^6^ CFU/mL for fungi). For gauzes and HEPA filters, COS was used for bacteria; COS and CAN2 for yeasts; COS and SDA for filamentous fungi. The media were chosen according to substrate geometry and microbial requirements. For catheters, the cylindrical segment was embedded in transparent molten TSA, a non-selective medium that allows the inhibition zone to be read uniformly around external and luminal surfaces. For the flat gauze and HEPA membranes placed onto pre-seeded plates, Columbia agar with 5% sheep blood (COS) was used as a rich, non-selective medium supporting the growth of all tested bacteria and providing optical contrast for halo reading; chromogenic CAN2 agar was added in parallel to confirm the species purity of the Candida suspensions, and Sabouraud Dextrose Agar (SDA), the elective medium for filamentous fungi, was used for Aspergillus and Penicillium. Assaying yeasts and molds on both COS and a selective/elective medium also verified that inhibition was independent of medium composition. Treated (TR) and untreated control (CTRL) segments were placed on inoculated plates and incubated at 37 °C. Inhibition halo diameter was recorded at 24 h, 7 days, and 15 days. Because the test articles are non-standard, three-dimensional device segments (catheter tubing, gauze meshes, and fiber-glass membranes) rather than standardized 6 mm paper disks, the halo was scored as a categorical presence/absence of a clearing zone within the limits of the SNV 195920-1992 contact method; quantitative antimicrobial efficacy was instead derived from CFU enumeration (below), which is independent of substrate geometry. Assessment was conducted in accordance with EUCAST and CLSI guidelines. A co-culture challenge test (*C. tropicalis* + *P. aeruginosa*) was included to evaluate activity maintenance in mixed microbial populations.

Quantitative microbial load reduction was determined by colony-forming unit (CFU) plate counts (triplicates) at each time point. Percentage reduction was calculated as:

(1) $$\frac{{CFU}_{CTRL}-{CFU}_{TR}}{{CFU}_{CTRL}}\times 100,$$

After 15 days, catheter segments were transferred to TSB for a 15-day recovery test (room temperature) to evaluate residual viable adherent flora.

### 2.6. Crystal Violet Biofilm Inhibition Assay

Biofilm formation kinetics were assessed in 96-well microtiter plates (100 μL inoculum/well; initial OD_600_ = 0.05) over 30 days [20,21]. Cultures were incubated at 37 °C for the first 24 h, then maintained at room temperature. Photometric readings were performed at 8 time points: 4, 12, 24, 48, and 72 h, and 7, 15, and 30 days. At each time point, planktonic cells were removed by three washes with distilled water; adherent biofilm was stained with 200 μL of 1% Crystal Violet (15 min, dark, room temperature), excess dye was removed by repeated washing, and bound dye was solubilized with 200 μL of 30% acetic acid (10–15 min agitation). Absorbance was measured at 630 nm.

The Biofilm Formation Index (%BFI), representing percentage inhibition relative to untreated controls, was calculated as:

(2) $$\%BFI=\left(1-\frac{{OD}_{test}-{OD}_{blank}}{{OD}_{control}-{OD}_{blank}}\right)\times 100,$$

Biofilm-producing phenotype classification followed the Stepanović criteria [22]: no production (OD ≤ ODc), weak (ODc < OD ≤ 2 × ODc), moderate (2 × ODc < OD ≤ 4 × ODc), or strong (OD > 4 × ODc), where ODc = mean OD of negative control + 3 standard deviations. As the Crystal Violet assay quantifies total adherent biomass rather than cell viability, it was paired in this work with viability-based readouts—the 15-day CFU recovery test in TSB and the post-assay replating of inhibition zones onto fresh COS agar (Section 3.2)—so that biomass and viable-cell endpoints could be interpreted jointly; Live/Dead and XTT metabolic-viability staining are foreseen as confirmatory assays in the planned in vivo-oriented phase.

### 2.7. Statistical Analysis

All protocols were performed in biological and technical triplicate. Data are expressed as mean ± standard deviation (SD). Statistical significance of differences was evaluated by Student’s t-test for quantitative variables. Normality of the %BFI and CFU-reduction distributions was verified by the Shapiro–Wilk test and homogeneity of variance by Levene’s test; where these assumptions were not met, the non-parametric Mann–Whitney U test was applied. For the simultaneous comparison of the ten isolates at each time point, *p*-values were adjusted for multiple comparisons with the Holm–Bonferroni correction and the Chi-square (χ2) test for categorical variables (biofilm phenotype distribution). All analyses and graphical representations were performed in R (v4.5, Windows environment). Significance threshold: *p* < 0.05.

## 3. Results

### 3.1. Treatment of the Three Clinical Substrates

**Figure 1:**
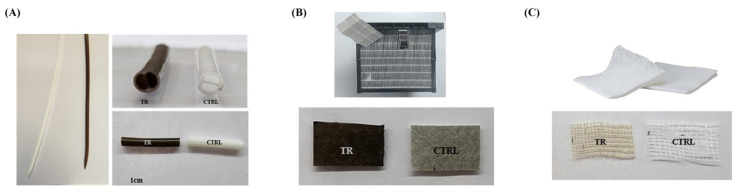
CTRL and TR samples of the three clinical substrates after treatment: (**A**) dual-lumen polyurethane venous catheters, (**B**) glass-fiber HEPA filter membranes, and (**C**) hydrophilic cotton gauze.

The color change observed after treatment, as shown in Figure 1, is indicative of successful substrate functionalization. This visual alteration is consistent with the formation of AgNPs on the material surface, resulting from the photochemical reduction of Ag^+^ ions to metallic silver.

Confirmatory characterization of the treated batch validated the deposition. EDX of functionalized catheters detected the characteristic silver peaks—absent in CTRL—at 0.54 wt% Ag in the as-prepared sample, decreasing only to 0.41 wt% after the 30-day intensive washing protocol, evidencing strong mechanical adhesion and low susceptibility to uncontrolled release (a barium signal from radiopaque BaSO_4_ in the polymer matrix was also recorded). SEM confirmed a largely uniform distribution of AgNPs on external and luminal surfaces; high-magnification imaging (50,000×) showed primary particles of ≈20 nm aggregating into 100–120 nm clusters, while TEM resolved single nanocrystals <2 nm displaying (111) silver lattice fringes. ICP-MS quantified a cumulative Ag^+^ release of 13.089 ppm over 30 days in PBS at 37 °C—a mean of ≈0.44 ppm·day^−1^—well below the U.S. EPA/ATSDR reference thresholds, confirming the “minimally diffusive” classification and supporting MDR (Regulation (EU) 2017/745) compliance for the standard 28–30-day catheter-use window. Morphologically, the functionalized surfaces appeared as a homogeneous brownish coating—consistent with the formation of metallic AgNPs—flattening the original micro-roughness of the polyurethane and uniformly decorating the cotton fibers and the glass-fiber mesh. Regarding per-substrate loading, silver content was quantified by EDX on the catheter as the reference substrate (0.54 wt% as-prepared; 0.41 wt% after 30-day washing). On the cotton gauze and the fiber-glass HEPA membrane, AgNP deposition was confirmed qualitatively by the characteristic EDX silver peaks and by SEM nanoparticle coverage, but the absolute per-substrate silver loading was not quantified in the present study. This is a direct consequence of the deposition chemistry: the precursor is a saturated ~4% silver-nitrate solution in anhydrous methanol, in which part of the salt is already undissolved, and the post-photoreduction rinsing removes further unreacted silver salt; consequently, only a fraction of the nominal silver—expected to differ between substrates according to their surface chemistry and area—is ultimately immobilized on each material, and this fraction was not determined. Substrate-resolved absolute loading (e.g., by acid-digestion ICP-MS, atomic absorption spectroscopy, or thermogravimetry) is identified as a dedicated quantification step for future work.

### 3.2. Antimicrobial Activity

#### 3.2.1. Venous Catheters

**Figure 2:**
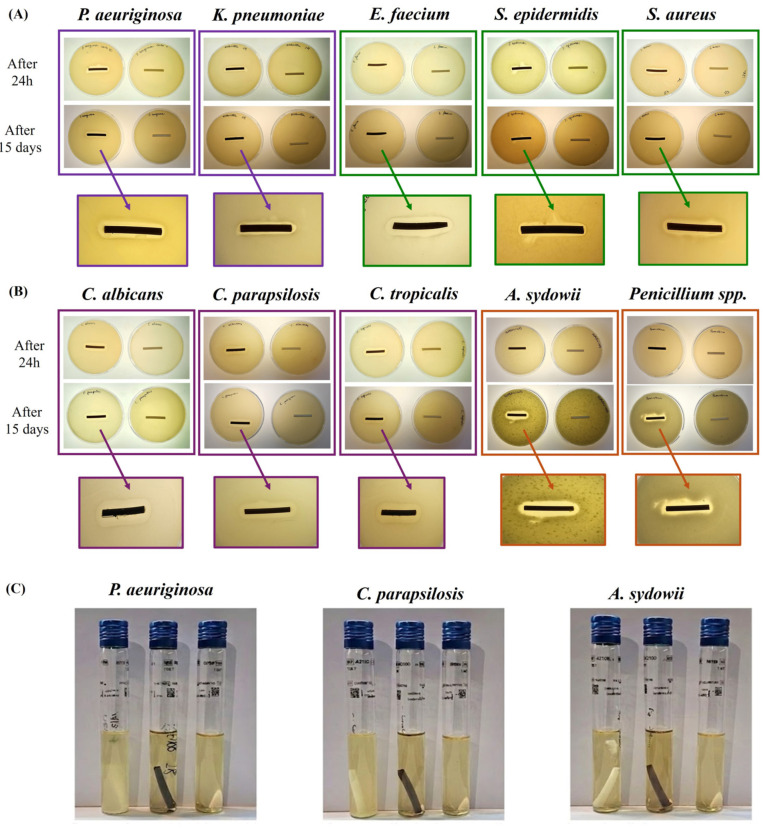
Antimicrobial activity of AgNP-functionalized polyurethane venous catheters (agar diffusion and recovery assays). (**A**) Agar diffusion assay on TSA inoculated with 0.5 McFarland bacterial suspensions of *P. aeruginosa*, *K. pneumoniae*, *E. faecium*, *S. epidermidis* and *S. aureus*. For each strain, the treated (TR) catheter segment (left) is compared with the untreated control (CTRL) at 24 h and 15 days; the lower row shows the magnified inhibition zone surrounding the treated segment at day 15. (**B**) Same assay on TSA inoculated with 0.5 McFarland fungal suspensions (*C. albicans*, *C. parapsilosis*, *C. tropicalis*, *A. sydowii*, *Penicillium* spp.). (**C**) Sessile-biomass eradication (recovery) test: catheter segments transferred to TSB after 15 days of agar incubation. Scale bar = 1 cm.

Agar diffusion assays on TSA plates inoculated with bacterial and fungal suspensions demonstrated clear inhibition halos around AgNP-treated catheters for all 10 tested strains at all three evaluation time points (24 h, 7 days, 15 days) (Figure 2A,B). Inhibition was observed from the first 24 h reading and maintained without attenuation over the full 15-day observation period. No inhibitory capacity was detected for CTRL segments, with unrestricted microbial growth reaching direct contact with the catheter surface. Quantitative CFU recovery showed a mean microbial load reduction of 98.7 ± 0.3% over 15 days (*p* < 0.002) compared to non-functionalized controls. This value is the mean of the percentage reductions computed individually for each of the 10 isolates (each in technical triplicate) and then averaged across the panel, not a single pooled figure; the corresponding per-isolate log_10_ CFU·mL^−1^ counts for treated and control segments. A co-culture challenge test (*C. tropicalis* + *P. aeruginosa*) confirmed that antimicrobial efficacy was unaffected by mixed microbial populations, demonstrating the robustness of the coating in complex infection scenarios. The 15-day recovery test in TSB liquid medium following agar incubation revealed complete absence of turbidity in tubes containing treated catheters, confirming no viable surface-adherent flora (Figure 2C). Conversely, CTRL catheter tubes exhibited marked turbidity; the *A. sydowii* control tube additionally showed mycelial filaments extending from the catheter surface into the culture medium, confirming active fungal colonization prevented by the AgNP coating [23].

#### 3.2.2. HEPA Filter Membranes

**Figure 3:**
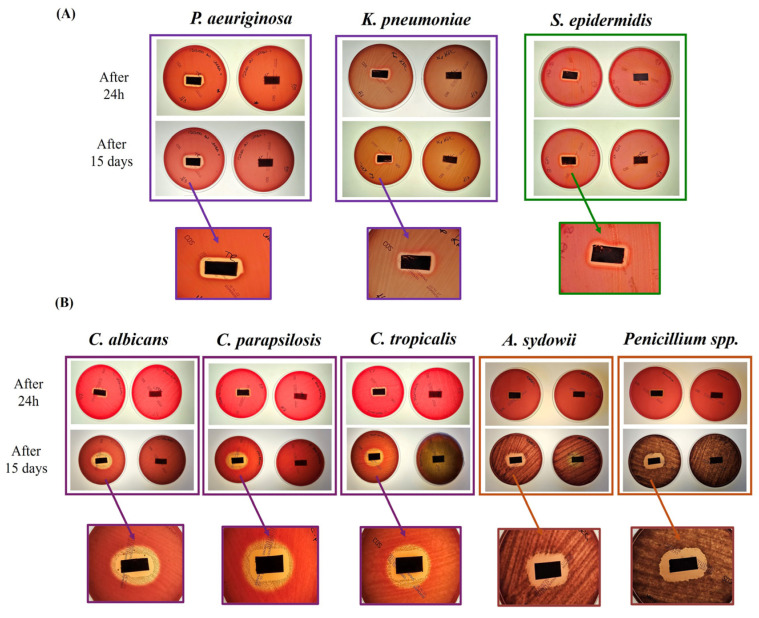
Antimicrobial activity of AgNP-functionalized HEPA filter membranes on COS. (**A**) Bacterial panel (*P. aeruginosa, K. pneumoniae*, *S. epidermidis*): inhibition halos surrounding treated (TR) membranes (left) versus uninhibited growth around untreated controls (CTRL) at 24 h and 15 days, with the magnified day-15 inhibition zone (lower row). (**B**) Fungal panel (*C. albicans*, *C. parapsilosis*, *C. tropicalis*, *A. sydowii*, *Penicillium* spp.); zenith (overhead) imaging shows the demarcation between mycelial expansion and the inhibition zone produced by the functionalized membrane. Scale bar = 1 cm.

AgNP-treated HEPA filter membranes showed significant inhibition halos on COS agar against all bacterial strains tested (*P. aeruginosa*, *K. pneumoniae*, *S. epidermidis*, *E. faecium*) and all fungal species (three *Candida* spp., *A. sydowii*, *Penicillium* spp.), with stable activity over 15 days (Figure 3A,B). Overhead (zenith) plate imaging of filamentous fungal assays clearly demonstrated the demarcation between fungal mycelial expansion and the surrounding inhibition zone produced by functionalized filters. Quantitative analysis documented a 99.8 ± 0.2% mean microbial load reduction over 15 days (*p* < 0.001).

While conventional HEPA filters physically capture ≥ 99.97% of particles ≥ 0.3 μm, the integrated AgNP biocidal activity neutralizes the viability of retained pathogens, reducing the risk of bioaerosol release during filter maintenance and preventing fungal colonization of the filtration membrane itself [24,25]. This synergistic functionality has clinical relevance for hematooncology units and transplant wards where preserved *Aspergillus* spp. conidia on filter surfaces represent a documented infection risk [25]. It should be noted that the present work employed an H14 glass-fiber membrane as an in vitro model of HEPA media to assess the biocidal function only; the aerodynamic performance of the functionalized medium—namely airflow resistance (differential pressure) and particle-filtration efficiency after AgNP deposition—was not evaluated here and constitutes a necessary step before integration into operating ventilation systems. This is addressed as a limitation and a defined objective of future work. No macroscopic alteration of the glass-fiber membrane (fragmentation, embrittlement, or discoloration beyond the expected AgNP coloration) was observed after the room-temperature methanol/UV functionalization, and SEM did not reveal evident fiber damage; nevertheless, a quantitative assessment of fiber integrity, pore-size distribution, and the associated pressure drop after coating is required and is planned together with the aerodynamic testing.

#### 3.2.3. Cotton Gauze Dressings

**Figure 4:**
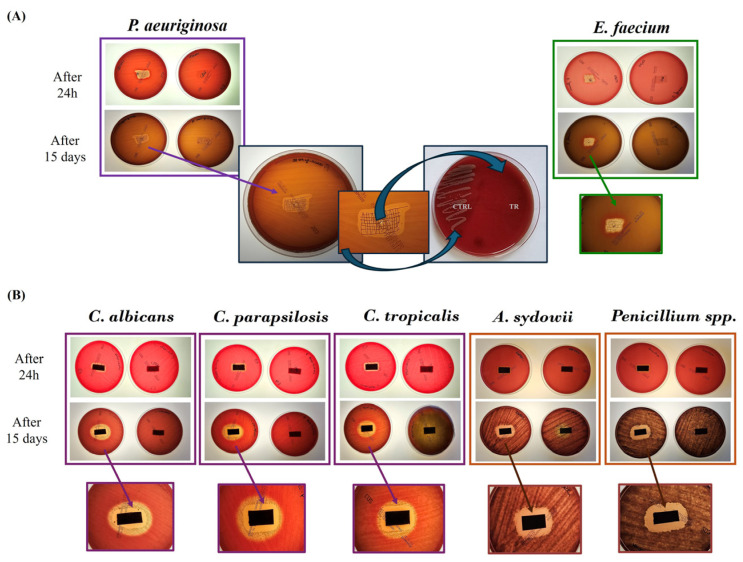
Antimicrobial activity of AgNP-functionalized cotton gauze dressings. (**A**) Bacterial assay on COS agar against *P. aeruginosa* and *E. faecium*: inhibition is evident both within the gauze mesh and in the surrounding perimeter for treated (TR) gauze (left) versus unrestricted growth for untreated controls (CTRL). The central inset illustrates the replating verification (subculture from the inhibition zone onto fresh COS agar confirming the absence of viable bacteria). (**B**) Fungal assay on chromogenic CAN2 / SDA and COS against the three *Candida* spp., *A. sydowii*, and *Penicillium* spp., confirming media-independent inhibition. Scale bar = 1 cm.

AgNP-functionalized gauzes demonstrated impressive inhibition halos against *P. aeruginosa* and *E. faecium* on COS agar plates, clearly visible both within the gauze mesh and in the surrounding perimeter zone (Figure 4A). CTRL gauzes permitted unrestricted bacterial growth. Replating from inhibition zones onto fresh COS agar after 15 days confirmed complete absence of viable bacteria (99.8 ± 0.1%, *p* < 0.0005), ruling out mere bacterio-static coloration artefacts; the observed opacity in inhibition areas was attributable exclusively to mucus and metabolite deposition, not to residual microbial viability. Candida species (*C. albicans*, *C. parapsilosis*, *C. tropicalis*) were simultaneously assayed on COS and CAN2 chromogenic agar, while *Aspergillus* spp. and *Penicillium* spp. were assayed on both COS and SDA, confirming inhibition independence from media composition and excluding enriched medium artefacts (Figure 4B). Gauze mechanical stability testing demonstrated retention of >92% antibacterial and antifungal activity after five PBS wash cycles at 37 °C [18,26]. Isotonic PBS at 37 °C was selected to approximate the physiological ionic strength and temperature of wound exudate, and five cycles were chosen to mirror the typical dressing-change frequency over a multi-day wound-care course; this protocol probes coating-substrate adhesion under repeated fluid challenge rather than reproducing the full complexity of an exuding wound bed. More clinically faithful media (simulated wound fluid, serum-containing exudate) and extended cycling are planned to consolidate this preliminary stability assessment.

### 3.3. Quantitative Biofilm Inhibition

**Figure 5:**
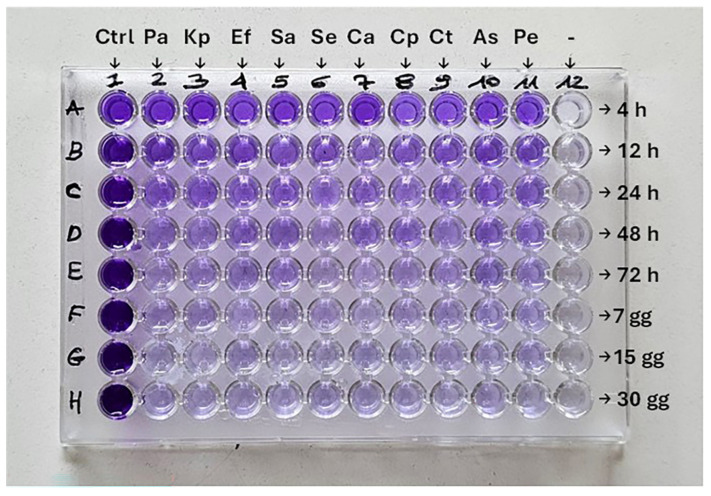
A representative stained microtiter plate across the 4 h–30-day time course.

Crystal Violet assay quantification of biofilm kinetics on AgNP-functionalized catheters confirmed the efficacy and rapidity of surface colonization prevention (Table 1; Figure 5). Biofilm inhibition was evident from the earliest time points: mean %BFI at 48 h was 92.0% for bacterial strains and 79.0% for fungal species. Efficacy consolidated progressively, reaching mean values of 97.7% (bacteria) and 97.4% (fungi) at day 15, with no statistically significant decay at day 30 (the slight biomass increases at day 30 being attributable to culture senescence and nutrient depletion rather than coating degradation). Among bacterial strains, *S. epidermidis* exhibited the highest susceptibility, with %BFI values of 97.5 ± 3.5% at 48 h and 98.5 ± 1.7% at 15 days, clinically significant given its role as the leading agent of CLABSI [27]. *E. faecium* reached the maximum inhibition of 98.6 ± 1.6% at day 15. Fungal species showed a characteristic initial kinetic delay, attributable to the structural robustness of the fungal cell wall (high chitin and β-glucan content), creating steric hindrance to ionic diffusion. However, this initial disparity was fully compensated by day 15, when *C. tropicalis* reached 98.0%, *C. parapsilosis* 97.9%, *Penicillium* spp. 97.3%, *A. sydowii* 96.7%, and *C. albicans* 97.0% inhibition. These data confirm that prolonged AgNP surface exposure allows nanoparticles to overcome the structural barriers of fungal pathogens [28,29].

## 4. Discussion

The outstanding microbiological performance observed across all three substrates directly correlates with the physicochemical characteristics of the surface photochemical coating and its kinetic properties, previously validated and reported by our research group [15,16] and confirmed by the literature [17,30]. Earlier investigations extensively validated the structural stability, surface morphology, silver distribution, and release kinetics of the functionalized materials through SEM, TEM, EDX, and ICP-MS analyses. To strengthen reproducibility and avoid relying solely on prior reports, confirmatory SEM/EDX/TEM imaging and ICP-MS release measurements were performed on the present treated batch and are reported in Section 3.1. These studies demonstrated a highly homogeneous and mechanically stable deposition of AgNPs, which remained firmly adherent to the substrate surfaces even after prolonged hydrodynamic stress conditions simulating clinical use [31]. Furthermore, microscopy analyses confirmed that the coating uniformly extended to both external and internal luminal surfaces of catheter devices, thereby ensuring protection at anatomical sites that are particularly vulnerable to microbial adhesion and biofilm development [32]. In parallel, previous biosafety assessments also conducted under simulated physiological conditions documented an extremely limited silver release profile [16]. This release rate was classified as “minimally diffusive” [22], remaining well below the no-observed-adverse-effect level (NOAEL) and the reference doses established by international regulatory agencies [30,33,34]. Such findings support the biocompatibility of the system and indicate that, under real clinical conditions, physiological blood dilution and normal clearance mechanisms would maintain systemic silver concentrations within accepted safety margins throughout the standard 28–30-day period of device utilization [30]. Therefore, the current microbiological findings should be interpreted within the framework of a coating system whose structural integrity and safety profile had already been comprehensively validated in previous studies [7,15,16,18,35].

In interpreting these mechanisms, this highly controlled, low-level release profile is advantageous as it ensures that localized contact-killing remains the predominant antimicrobial pathway; this fundamentally undermines primary bacterial silver-resistance mechanisms, such as the sil operon or cus efflux pump systems, which successfully detoxify free Ag+ ions but are entirely ineffective against direct nanoparticle surface contact [36]. Consequently, this pleiotropic, ROS-mediated contact action explains the persistent broad-spectrum bactericidal and fungicidal efficacy observed over 15 days against the entire 10-strain MDR panel [11,12]. We emphasize that, in the present study, this mechanism is inferred from the phenotypic efficacy data combined with the well-established mode of action of AgNPs documented for this validated photochemical platform, rather than demonstrated by direct mechanistic assays; dedicated experiments—intracellular ROS quantification, membrane-integrity/permeability assays and ultrastructural (TEM) analysis of treated cells—are planned to substantiate these pathways and are acknowledged as a limitation of the current work. Crucially, the massive biofilm inhibition rates quantified at day 15, exceeding 97% for *S. epidermidis* and 96% for fungal species, carry immense clinical weight. Given that coagulase-negative staphylococci, primarily *S. epidermidis*, are the leading etiological drivers of central line-associated bloodstream infections (CLABSIs) in intensive care settings [27], these findings demonstrate that the uniform AgNP coating effectively blocks microbial colonization and subsequent lattice maturation at the device-tissue interface. While an initial kinetic lag in fungal inhibition (78–80% at 48 h) was observed compared to bacteria (>88%), this is attributable to the structural rigidity and high chitosan/β-glucan content of the fungal cell wall creating a temporary steric hindrance to initial ionic diffusion [28,29]; the subsequent convergence of inhibition rates by day 15 proves that the sustained contact-killing mechanism successfully overcomes this structural barrier over time.

This robust biocidal efficacy translates directly into targeted solutions for major unmet medical needs. By neutralizing airborne conidia of *Aspergillus* and *Penicillium* spp., the functionalized HEPA membranes transition from passive physical barriers into active antimicrobial surfaces, mitigating the risk of opportunistic bioaerosol reservoirs during filter maintenance or hospital renovations [24,25], a critical advancement for hematooncology and transplant units where invasive pulmonary aspergillosis carries devastating mortality rates of 30–50% [25]. Similarly, the functionalized gauzes achieved a >99% CFU reduction against *P. aeruginosa* and *E. faecium* while preserving over 92% of their activity, confirming their suitability for clinical wound care [18,26]. Furthermore, in accordance with the principles of Planetary Health and One Health, the absence of leaching nature of this system prevents the ecotoxicological contamination of hospital effluents, thereby reducing horizontal gene transfer (HGT) selection pressure in the environmental microbiome [7] while offering substantial institutional cost savings per prevented infection episode.

## 5. Conclusions

In conclusion, this study establishes the photochemical deposition of silver nanoparticles as a highly effective, primary-prevention paradigm against healthcare-associated infections across polyurethane catheters, cotton gauzes, and HEPA membranes. Crucially, the strong and consistent microbiological results achieved by these functionalized devices highlight their clinical potential, demonstrating how precision nanotechnology can successfully bypass classical antimicrobial resistance (AMR) pathways to eradicate high-priority threats.

The engineered coating exerted persistent, broad-spectrum antimicrobial and antifungal activity over 15 days against a highly challenging 10-strain multidrug-resistant (MDR) clinical panel. Mean microbial load reductions reached high levels, exceeding 98.7% on catheters and 99.8% on both HEPA membranes and cotton gauzes. Furthermore, the functionalized surfaces markedly reduced bacterial biofilm formation, achieving inhibition of over 96% at day 15 across all tested strains. This reduction is noteworthy, as it was achieved against critical, pan-resistant pathogens, including carbapenem-resistant *Klebsiella pneumoniae*, MDR *Pseudomonas aeruginosa*, and azole-resistant *Candida* spp., which routinely evade conventional clinical therapies.

By translating multi-target biological mechanisms into predictable therapeutic outcomes, these functionalized biomaterials offer promising possibilities for infection control. They transcend traditional barrier coatings to emerge as concrete, scalable assets within modern Antimicrobial Stewardship, Circular Nanomedicine, and Planetary Health frameworks. Pending further biological safety characterization per ISO 10993 standards [37] and prospective clinical validation, these silver-functionalized medical devices represent a structurally innovative, bio-sustainable, in vitro candidate strategy that warrants further translational evaluation within the global effort against the escalating AMR crisis. Specifically, prior to clinical translation, the system will undergo the ISO 10993 biological-evaluation series—including in vitro cytotoxicity on HUVEC endothelial cells, hemolysis testing on erythrocytes, and sub-chronic systemic-toxicity and long-term genotoxicity studies—followed by in vivo biocompatibility assessment under dynamic physiological (blood-flow and plasma-protein) conditions.

## Figures and Tables

**Table 1:** %BFI values at 48 h, 7 days and 15 days for the ten clinical isolates. The complete eight-time-point kinetic dataset (4, 12, 24, 48 and 72 h, and 7, 15 and 30 days), of which the present table reports representative values, is plotted in Figure 5.

Microorganism	%BFI at 48 h	%BFI at 7 Days	%BFI at 15 Days
*Pseudomonas aeruginosa*	90.4 ± 4.8	98.0 ± 1.8	97.8 ± 2.0
*Klebsiella pneumoniae*	93.6 ± 4.6	95.7 ± 1.3	95.8 ± 1.8
*Enterococcus faecium*	88.1 ± 3.2	98.3 ± 1.2	98.6 ± 1.6
*Staphylococcus aureus*	90.4 ± 5.1	97.6 ± 2.2	97.6 ± 2.1
*Staphylococcus epidermidis*	97.5 ± 3.5	98.4 ± 1.7	98.5 ± 1.7
*Candida albicans*	78.6 ± 5.8	90.1 ± 2.3	97.0 ± 2.4
*Candida parapsilosis*	79.0 ± 5.1	91.6 ± 2.2	97.9 ± 1.1
*Candida tropicalis*	80.7 ± 4.7	92.6 ± 1.9	98.0 ± 2.4
*Aspergillus sydowii*	77.6 ± 3.3	89.8 ± 2.1	96.7 ± 1.3
*Penicillium* spp.	79.4 ± 4.2	92.4 ± 1.6	97.3 ± 1.1

## Data Availability

The original contributions presented in this study are included in the article. Further inquiries can be directed to the corresponding author.

## Abbreviations

The following abbreviations are used in this manuscript:

HAIs	Healthcare-associated infections
WHO	World Health Organization
ECDC	European Centre for Disease Prevention and Control
CAUTI	Catheter-Associated Urinary Tract Infections
VAP	Ventilator-Associated Pneumonia
SSI	Surgical Site Infections
CLABSI	Central Line-Associated Blood Stream Infections
AMR	Antimicrobial Resistance
HGT	Horizontal Gene Transfer
AgNPs	Silver Nanoparticles
ROS	Reactive Oxygen Species
AgNO_3_	Silver Nitrate
CFU	Colony-Forming Unit
SD	Standard Deviation
